# The Effect of Ginger (*Zingiber officinalis*) and Artichoke (*Cynara cardunculus*) Extract Supplementation on Functional Dyspepsia: A Randomised, Double-Blind, and Placebo-Controlled Clinical Trial

**DOI:** 10.1155/2015/915087

**Published:** 2015-04-14

**Authors:** Attilio Giacosa, Davide Guido, Mario Grassi, Antonella Riva, Paolo Morazzoni, Ezio Bombardelli, Simone Perna, Milena A. Faliva, Mariangela Rondanelli

**Affiliations:** ^1^Department of Gastroenterology, Policlinico di Monza, 20900 Milan, Italy; ^2^Section of Biostatistics, Neurophysiology and Psychiatry, Department of Brain and Behavioral Sciences, University of Pavia, 27100 Pavia, Italy; ^3^Research and Development Unit, Indena, 20139 Milan, Italy; ^4^Section of Human Nutrition, Department of Public Health, Experimental and Forensic Medicine, University of Pavia, Azienda di Servizi alla Persona, 27100 Pavia, Italy

## Abstract

*Objective.* Functional dyspepsia (FD) is a frequent clinical finding in western world. The aim of this study is to compare the efficacy of a ginger and artichoke supplementation versus placebo in the treatment of FD.* Methods.* A prospective multicentre, double blind, randomized, placebo controlled, parallel-group comparison of the supplement and placebo over a period of 4 weeks was performed. Two capsules/day were supplied (before lunch and dinner) to 126 FD patients (supplementation/placebo: 65/61).* Results*. After 14 days of treatment, only supplementation group (SG) showed a significant amelioration (SG: *α*_S_ = +1.195 MCA score units (u), *P* = 0.017; placebo: *α*_P_ = +0.347 u, *P* = 0.513). The intercept (*α*) resulted to be significantly higher in SG than in placebo (*α*_S_ − *α*_P_ = +0.848 u, *P* < 0.001). At the end of the study, the advantage of SG versus placebo persists without variation (*β*_S_ − *β*_P_ = +0.077 u, *P* = 0.542). In SG, a significant advantage is observed for nausea (*β*_S_ − *β*_P_ = −0.398 u, *P* < 0.001), epigastric fullness (*β*_S_ − *β*_P_ = −0.241, *P* < 0.001), epigastric pain (*β*_S_ − *β*_P_ = −0.173 u, *P* = 0.002), and bloating (*β*_S_ − *β*_P_ = −0.167 u, *P* = 0.017).* Conclusions*. The association between ginger and artichoke leaf extracts appears safe and efficacious in the treatment of FD and could represent a promising treatment for this disease.

## 1. Introduction

Functional dyspepsia (FD) is a frequent clinical finding in the Western world and is a major cause of morbidity and economic loss in the community [1–5]. The mostly used definition of FD refers to chronic or recurrent pain or discomfort centered in the upper abdomen in the absence of any known structural cause and without features of irritable bowel syndrome or of gastroesophageal reflux [5]. In FD symptoms are frequently correlated to meals and may include abdominal pain, bloating, early satiety, fullness, belching, and nausea [5]. The most recent definition and classification of functional dyspepsia is known as Rome III criteria for the diagnosis of functional dyspepsia. Anyhow various authors and in particular a recent paper of Ford et al. state that the Rome III criteria for the diagnosis of functional dyspepsia are not superior to previous definitions [6].

The prevalence of FD has been noted to vary between 11% and 29.2% of adult population with relevant geographical variation and the impact of FD on patients and health care services has been shown to be considerable [7]. Various pathophysiological mechanisms may account for the etiology of FD, such as impaired meal induced relaxation of the proximal stomach, visceral hypersensitivity to distension, gastric motor abnormalities, and disturbed central nervous function [8]. This multifactorial and poorly defined pathogenesis has hampered efforts to develop effective treatments in most of cases of FD [9], and its optimal clinical management remains a subject of considerable debate [10].

Great attention has always been paid to the antinausea and antivomiting effect of the extracts of ginger and to their activity on gastric motility [11, 12]. Artichoke leaf extracts (ALE) have been used since long time, in traditional medicine, to treat dyspepsia, and in 2003 Holtmann et al. [13] confirmed this effect in patients with FD.

The aim of this controlled double-blinded study is to verify the efficacy of the combination of ginger and artichoke extracts in the treatment of functional dyspepsia.

## 2. Subjects and Methods

### 2.1. Patients

Adult male and female patients with functional dyspepsia and with age ranging from 18 to 70 years were selected for this study at the “Santa Rita Institute” of Vercelli and at the “Santa Margherita Institute” of Pavia. Functional dyspepsia was defined according to the consensus statement published by Talley et al. [5, 14, 15]. This definition identifies patients with upper abdominal pain or discomfort that is an unpleasant sensation characterized by one or more of the following symptoms: early satiety, postprandial fullness, bloating, and nausea for at least 3 months during last year, without an identifiable underlying structural or biochemical motivation [5]. According to Rome II criteria, patients with functional dyspepsia (FD) were classified into three subgroups: ulcer-like FD, dysmotility FD, and unspecified or nonspecific functional dyspepsia [5]. Patients with relevant gastroesophageal reflux symptoms (retrosternal pain, burning, or regurgitation) as well as patients with clear evidence of irritable bowel syndrome were not included. Patients under treatment with pharmacological substances that could influence the gastrointestinal system, such as prokinetics, ursodeoxycholic acid (UDCA), nonsteroidal anti-inflammatory drugs (NSAIDs), cholagogues, proton-pump inhibitors, and H2 blockers, were asked to interrupt this treatment for one month before starting the study treatment. Moreover, patients with previous diagnosis of cancer or with previous surgery of the upper gastrointestinal tract or of the biliopancreatic system (except for cholecystectomy) and patients with active HP infection or with gastric or duodenal ulcer, as well as pregnant women, were excluded. Prior to enrolment in the trial (within one month before randomization), a physical examination, laboratory testing (full blood count, sedimentation rate, liver function tests, fasting blood glucose, and creatinine), abdominal ultrasound examination, and upper gastrointestinal endoscopy were performed in order to exclude the presence of structural or biochemical causes of dyspepsia.

### 2.2. Study Design

A prospective, multicentre, double-blind, randomized, placebo controlled, parallel-group comparison of the supplement (ginger and artichoke leaf extracts) and placebo over a period of 4 weeks was performed. The comparison with placebo has been chosen in absence of an evidence based choice treatment of functional dyspepsia. This choice has been done in agreement with the Helsinki Declaration of patient's rights [16].

The primary outcome of the study was the overall change of intensity of functional dyspepsia as defined by patient's rating after two and four weeks of intervention with the ginger and artichoke supplement or with placebo. Patient's ratings were ascribed to one of four categories (0: not improved or worsened, 1: slightly improved, 2: markedly improved, and 3: completely improved). The severity of each of the following six dyspeptic symptoms, epigastric fullness, bloating, early satiety, nausea, vomiting, and epigastric pain, was used as secondary outcome. The self-reported patient's rating of the intensity of dyspeptic symptoms was rated on a four-point Likert scale ranging from absence of symptoms (0 points) to severe symptoms (3 points) [17].

### 2.3. Visits

Four visits were scheduled. Visit 1 was a screening visit with signature of the written consent; visit 2 was a baseline visit 28 days after visit 1 with randomization of patients matching inclusion and exclusion criteria and start of treatment. Two additional visits were planned, respectively, after two (visit 3) and four (visit 4) weeks of intervention. At visits 3 and 4 the overall change of functional dyspepsia and the intensity of individual dyspeptic symptoms were assessed. Ethics committee approval was obtained prior to commencing the study (May 16, 2012).

### 2.4. Dietary Supplement

Hard gelatin capsules, each one containing 100 mg of artichoke and 20 mg of ginger standardized extracts, were used (ProDigest, WO2010/083968): each component was characterized by an elevated stability. Both extracts were prepared using extraction procedures suitable for ingredients of the health food market. The artichoke extract is characterized by a high content of caffeoylquinic acid (HPLC >20%) and flavonoids (HPLC > 5%) along with cynaropicrin (>5%); the ginger extract was characterized by a high content of total gingerols (25.0–30.0% total gingerols).

The enrolled patients were randomized to* Zingiber officinalis* and* Cynara cardunculus* complex or to placebo. Identical and odorless capsules for each treatment group (intervention and placebo) were assigned according to a randomization code. Both products (active supplement and placebo) were provided free of charge by Indena (Milan) in packaging specifically designed for this study.

All patients were instructed to take 1 capsule immediately before starting lunch and dinner. During 30 days before the beginning of the study and during the 28 days of intervention treatment all patients were asked to avoid the use of prokinetic and antisecretory (H2 receptor inhibitors and proton pump inhibitors) drugs. All patients were also asked to limit the consumption of alcohol beverages (less than or equal to two drinks per day) and to follow a “prudent diet,” avoiding fried or spicy foods, sauces, stews, and gravies.

### 2.5. Adverse Events

Unwanted adverse events, tolerability, vital signs, and clinically significant modifications in laboratory values were monitored.

### 2.6. Randomization

Patients who met the admission criteria and who signed the informed consent to the study have been assigned consecutive and growing numbers of randomization, starting with number 1, according to a 1 : 1 ratio. Investigators were blinded to the randomization table.

### 2.7. Sample Size

Considering alpha = 0.05 and a study power of 90% and assuming, on the basis of the results of a previous study of Holtmann et al. [13], a mean sum-score for the primary outcome of 2 in the placebo group and an expected advantage for the intervention group of 0.9 with an estimated standard deviation of 1.5, it was calculated that 120 patients (60 in the experimental group and 60 in the placebo group) were needed to test properly the difference between the two treatment groups.

### 2.8. Statistical Analysis

To compare supplementation (S) and placebo (P) we assessed primary and secondary outcome trends by latent curve models (LCMs) for repeated measures using a structural equations approach [18]. Firstly, we performed a preprocessing phase in which we linearized the ordinal outcome data, making it numerical, by multiple correspondence analysis (MCA) quantifications [19]. In order to compare all the outcomes we normalized the quantifications in respect of the first one, that is, setting the quantification of the initial category equal to zero. Secondly, in LCM phase, for each quantified outcome, we estimated two parameters, the intercept (*α*), the average baseline measurement of MCA severity score at the first time point, and the slope (*β*), the average trend (trajectory) over time of the MCA severity scores. Estimates were adjusted for dyspepsia typology covariate (ulcer-like, dysmotility-like, and unspecified functional dyspepsia) and for baseline values of symptom severity scores. The latter adjustment is performed to manage imbalances of symptom severities across treatments. The planned sample size (*n* = 120) was sufficient for fitted LCMs based on the ratio of sample size to number of parameters to be estimated in structural equation models (SEM) [20]. We considered two-sided *P* values less than 0.05 to be statistically significant. We used aspect and lavaan [21] packages of R software [22] for MCA scaling and LCM analysis, respectively.

## 3. Results

### 3.1. Descriptive Data

**Figure 1:**
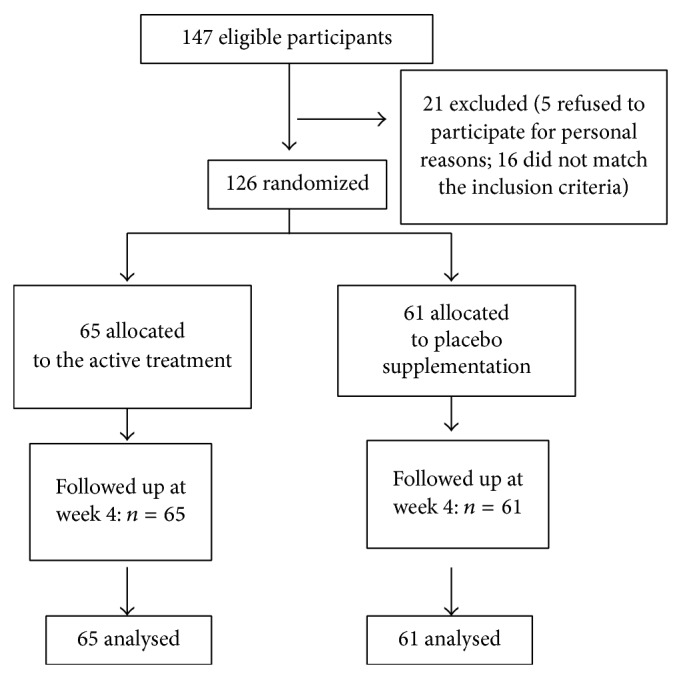
Flow diagram of a trial of supplementation with a combination of two highly standardized extracts from* Zingiber officinale* and* Cynara cardunculus* in the treatment of functional dyspepsia. The diagram includes the number of patients analyzed for the main outcome (effect on functional dyspepsia).

The statistical analysis of the baseline descriptive data shows that the randomization has been correctly operated and that the intervention and placebo groups are homogenous as age (45.8 versus 48.0 years) and the male/female ratio (19/46 versus 20/41) are concerned (Table 1). Table 1 shows also the homogenous allocation by dyspepsia typology. In Figure 1 the flow diagram of the trial is reported.

Table 2 shows that the supplementation with ginger and artichoke extracts is efficacious in the short-term treatment of FD (within 14 days), and afterwards it is maintained until the 28th day of intervention. The intervention group shows treatment efficacy, measured by raw scores (0–3), in 86.2% of cases after 28 days of active supplementation, with marked reduction of dyspepsia intensity (grades 2 + 3) in 63.1% of the treated cases, while 52.5% of the placebo group patients showed a positive effect of placebo and only 24.6% of the placebo treated patients had a marked reduction of symptoms (grades 2 + 3). The percentage difference in the global response, between the intervention product and placebo, was 33.7%.

### 3.2. Multiple Correspondence Analysis (MCA) Preprocessing Step

The MCA preprocessing step, performed on each outcome, returned a correct but nonequidistant “matching” of quantifications with the initial score (0–4) of the ordinal responses. Only baseline values of “early satiety” did not return the expected ordinal “matching,” but this finding is not statistically significant (Table 3). Thus, the MCA quantifications were considered as numerical points of the ordinal outcomes in the modelling step (LCM).

### 3.3. Primary Outcome

**Figure 2:**
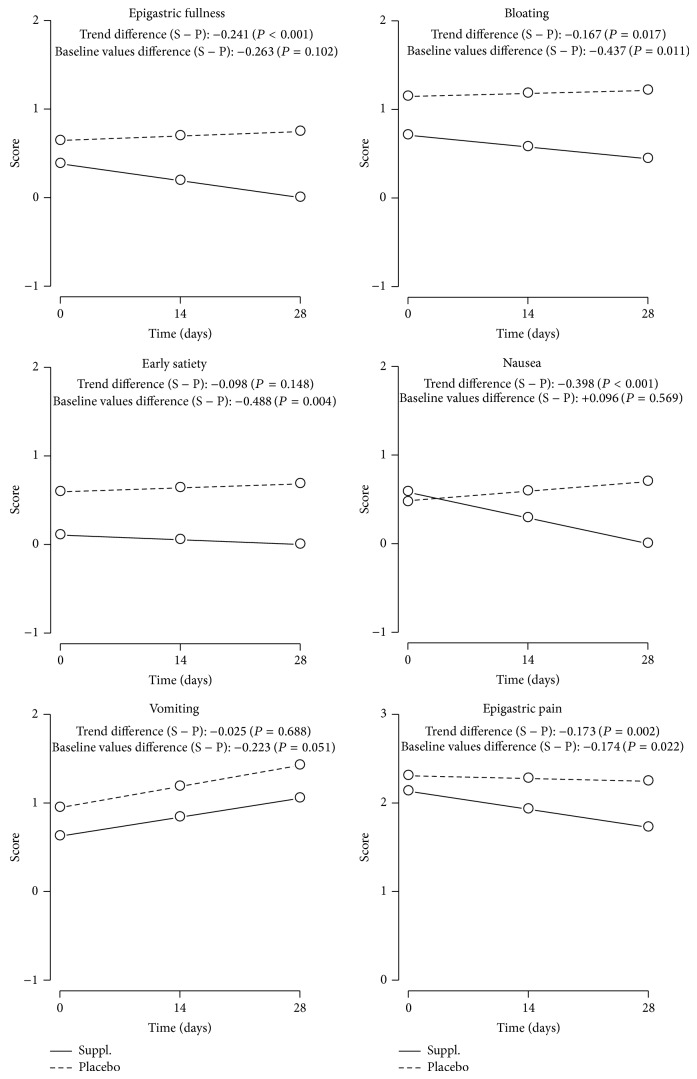
Trend of secondary outcomes.

The primary outcome of the study was the overall change of intensity of functional dyspepsia as defined by patient's rating. After two out of four weeks of intervention, according to the latent curve models (LCMs) step (see Table 3 and Figure 2), only supplementation group showed a significant amelioration on the MCA severity scale (of range 0–3) at 14 days, measured by intercept parameter of LCM (supplementation group: *α*_S_ = +1.195 units, *P* = 0.017; placebo group: *α*_P_ = +0.347 units, *P* = 0.513). In fact the intercept was significantly higher in patients treated with supplementation than in the placebo group (*α*_S_ − *α*_P_ = +0.848 units, *P* < 0.001).

This finding reveals that the supplementation treatment has a greater efficacy than placebo to treat functional dyspepsia, when data are compared at 14 days after treatment. When the intensity of functional dyspepsia at the end of the study (28 days) is compared with values at 14 days, measured by slope parameter of LGM, no variation was observed both over time (supplementation group: *β*_S_ = −0.181 units, *P* = 0.619; placebo group: *β*_P_ = −0.258 units, *P* = 0.504) and across groups (*β*_S_ − *β*_P_ = +0.077 units, *P* = 0.542).

### 3.4. Secondary Outcomes

Accounting for secondary outcomes, firstly, it must be noted that all LCM slopes-*β*_*s*_ (i.e., the score variation across time), excluding vomiting (*β*_*s*_ = +0.214, *P* = 0.056), are negative, decreasing in intensity, while all placebo-*β*_*s*_, in opposite, are positive. This finding indicates the greater efficacy of the new treatment as compared to placebo on five out of six specific symptoms (Table 4). The statistical analysis shows a significant intensity score reduction for nausea (*β*_*s*_ = −0.290, *P* = 0.030) and epigastric pain (*β*_*s*_ = −0.204, *P* = 0.036) over the observation time in the intervention group. In the placebo group the slope shows an increase of intensity for all the considered symptoms with a statistically significant worsening for vomiting.

This is confirmed by the slope difference between the placebo and intervention groups showing a significant advantage for the intervention group as far as nausea (*β*_S_ − *β*_P_ = −0.398, *P* < 0.001), epigastric fullness (*β*_S_ − *β*_P_ = −0.241, *P* < 0.001), epigastric pain (−0.173, *P* = 0.002), and bloating (*β*_P_ − *β*_S_ = −0.167, *P* = 0.017) are concerned (Table 4 and Figure 2).

## 4. Discussion

The main result of this study is that the supplementation with ginger and artichoke extracts is efficacious in the short-term treatment of FD. This effect appears to be statistically significant when compared to placebo. It is interesting to note that the efficacy appears quickly, that is, within 14 days, and afterwards it is maintained until the 28th day of intervention. The intervention group shows treatment efficacy in 86.2% of cases after 28 days of supplementation, with marked reduction of dyspepsia intensity (grades 2 + 3 of the considered scale) in 63.1% of the treated cases, while only 52.5% of the control group patients showed a positive effect of placebo and only 24.6% of the placebo treated patients had a marked reduction of symptoms (grades 2 and 3).

Our results show the advantage of the supplementation, as compared to placebo, with a significant amelioration of 0.85 units on the MCA severity scale (of range 0–3) at 14 days. This result is adjusted for baseline symptoms and typologies of dyspepsia, and it persists until the end of the study (28th day). On raw data, the percentage difference between the intervention product and placebo approached 34%. This therapeutic gain is greater than what has been observed in previous studies with antisecretory and gastrokinetic drugs [23], as well as with artichoke extracts [13]: in all these cases the advantage was in the range of 15%. Therefore, it seems that the association between ginger and artichoke extracts may increase the treatment efficacy on FD as compared to what was observed with artichoke extract alone or with antisecretory and gastrokinetic drugs.

The mechanisms involved in the pathophysiology of FD are multifactorial. As a matter of fact, a number of potential abnormalities have been reported in patients with FD including impaired fundic accommodation, gastric hypersensitivity to distention, abnormal duodenojejunal motility, duodenal motor and sensory dysfunction, duodenal hypersensitivity to lipids or acid, and* Helicobacter pylori* infection [24]. In the present study the highest prevalence of FD subtype was represented by dysmotility-like FD and unspecified FD, while ulcer-like FD was present in very few cases of both the intervention and placebo groups, as shown in Table 1. Therefore, a prevalence of symptoms related to motility disorders was observed in the recruited patients. Most studies in animals have demonstrated that ginger root extracts increase gastric emptying and gastrointestinal transit [25]. Micklefield et al. demonstrated a significant increase of the interdigestive motility after intervention with ginger extracts and Wu et al. showed that ginger accelerates gastric emptying and stimulates antral contractions in healthy volunteers [11, 26]. Animal emesis models likewise have shown reduced emesis with the administration of ginger. Gingerols and shogaols seem to be the active components [27].

Nausea, vomiting, and hypomotility involve a temporary dysfunction of the complex integrated network of cholinergic M3 and serotonergic 5-HT3/5-HT4 receptors. In this respect, major chemical constituents of the ginger roots lipophilic extracts such as [6]-gingerol, [8]-gingerol, [10]-gingerol, and [6]-shogaol have been shown in several experimental models to modulate with a differentiated potency all these receptors. In particular, the capacity of ginger to reduce nausea and eventually vomiting seems to correlate with the effectiveness of these active ingredients to weakly inhibit M3 and 5-HT3 receptors. On the contrary, 5-HT4 receptors, which also play a role in gastroduodenal motility, do not seem to be involved in the effects of these compounds [11, 12].

Artichoke leaf extracts (ALE) have been used since long time, in traditional medicine, to treat dyspepsia and in 2003 Holtmann et al. [13] confirmed this effect in patients with FD. ALE increase bile flow and exert hepatoprotective [28], serum cholesterol lowering [29], and antioxidant and antispasmodic effects [30–32]. The bitter compounds of ALE and particularly cynaropicrin are responsible for the digestive beneficial effects [33, 34]. Holtmann et al. [13] showed that ALE were significantly better than placebo in reducing symptoms and improving the disease-specific quality of life in patients with functional dyspepsia. Anyhow the present study shows that the association of ginger extracts and ALE increases the efficacy on functional dyspepsia treatment with a 16.9% advantage as compared to the data found by Holtmann et al. [13] with ALE alone.

Of great interest appears the evaluation of the effect of ginger and artichoke supplementation on specific symptoms of functional dyspepsia. In this study the intervention was associated with a reduction of severity of epigastric pain, epigastric fullness, nausea, bloating, and early satiety: this decrease appears statistically significant for nausea and epigastric pain, over the observation time of 28 days. The only symptom which did not change was vomiting. On the contrary, in the placebo group, the statistical analysis shows that all symptoms have an increasing slope, which means an increase of symptom intensity with a statistically significant worsening for vomiting.

Considering the entity of the efficacy on the secondary outcomes, the supplementation shows a greatest efficacy on nausea, followed by a positive effect on epigastric fullness, epigastric pain, and bloating, after statistical adjustment for type of dyspepsia. The effect on nausea and on epigastric fullness could mainly be due to the ginger component and to its activity on gastric motility: this confirms what was previously observed in other clinical settings such as in nausea associated with pregnancy, chemotherapy, and motion sickness [12, 35–37].

The effects on bile secretion that have been found in previous trials with artichoke extracts [38] may partially contribute to our results. The increase in bile acid secretion, observed after supplementation with ALE, is suitable to accelerate gastrointestinal transit and thus may alleviate bloating and fullness. The well-known antispasmodic feature of ALE may also increase both effects [38].

The treatment with the ginger and ALE supplement used in this study did not show any relevant side effect. This observation is of great importance when compared with the critical role of traditional prokinetic drugs such as domperidone, levosulpiride, or metoclopramide, frequently used in FD therapy. The treatment with these drugs is frequently associated with neurologic or endocrinologic side effects [39]. In addition to this, a severe warning has been reported for domperidone treatment due to the sudden risk of cardiac death observed at doses of more than 30 mg per day in the elderly [40–43].

In conclusion, the association between ginger and artichoke leaf extracts appears efficacious in the treatment of functional dyspepsia and could represent a promising and safe treatment strategy for this frequent disease, even though additional studies are needed to confirm these results.

## Figures and Tables

**Table 1:** Baseline description of the sample.

	Supplementation group	Placebo group
Randomized patients	65	61
Male/female	19/46	20/41
Age (yr) (mean ± SD)	45.85 ± 16.61	48.05 ± 17.02
Type of functional dyspepsia (FD)		
Ulcer-like FD	5	6
Dysmotility-like FD	39	33
Unspecified FD	21	22

Significant differences are absent when the intervention and the placebo groups are compared.

**Table 2:** Frequencies of functional dyspepsia over time across treatment.

	Score	Supplementation group (*n* = 65)	Placebo group (*n* = 61)
T1 (after 14 days)	0	10 (15.4%)	26 (42.6%)
	1	26 (40%)	27 (44.3%)
	2	25 (38.5%)	8 (13.1%)
	3	4 (6.1%)	0 (0)

T2 (after 28 days)	0	9 (13.8%)	29 (47.5%)
	1	15 (23.1%)	17 (27.9%)
	2	21 (32.3%)	13 (21.3%)
	3	20 (30.8%)	2 (3.3%)
	2 + 3	41 (63.1%)	15 (24.6%)
	1 + 2 + 3	56 (86.2%)	32 (52.4%)

**Table 3:** Frequency distributions and MCA quantifications^*^ of the primary and secondary outcomes.

		T0 (baseline)				T1 (after 14 days)				T2 (after 28 days)			
		0	1	2	3	0	1	2	3	0	1	2	3
Functional dyspepsia (primary outcome)	S	—	—	—	—	10	26	25	4	9	15	21	20
	P	—	—	—	—	26	27	8	0	29	17	13	2
	Q	—	—	—	—	**0**	**1.23**	**2.52**	**3.28**	**0**	**0.98**	**1.86**	**2.78**

Epigastric fullness	S	22	5	16	22	30	21	9	5	40	16	5	4
	P	12	14	13	22	14	21	17	9	17	22	12	10
	Q	**0**	**1.45**	**1.87**	**2.52**	**0**	**1.61**	**2.25**	**2.61**	**0**	**1.69**	**2.13**	**2.46**

Bloating	S	30	7	11	17	37	14	12	2	46	8	9	2
	P	13	17	19	12	15	24	18	4	19	20	19	3
	Q	**0**	**1.61**	**2.08**	**2.37**	**0**	**1.64**	**2.27**	**2.47**	**0**	**1.74**	**2.13**	**2.40**

Early satiety	S	52	2	6	5	57	5	2	1	61	2	2	0
	P	36	13	4	8	38	17	5	1	41	14	5	1
	Q	**0**	**2.24**	**2.14**	**2.14**	**0**	**2.19**	**2.57**	**2.75**	**0**	**2.42**	**2.75**	**2.91**

Nausea	S	21	7	9	28	32	21	9	3	51	6	7	1
	P	22	12	18	9	23	25	10	3	27	18	11	5
	Q	**0**	**1.79**	**2.17**	**2.18**	**0**	**1.67**	**2.39**	**2.59**	**0**	**1.86**	**2.19**	**2.37**

Vomiting	S	61	1	2	1	62	3	0	0	63	2	0	0
	P	48	9	1	3	53	6	2	0	52	7	2	0
	Q	**0**	**2.33**	**3.58**	**3.59**	**0**	**2.52**	**6.27**	**—**	**0**	**2.52**	**6.28**	**—**

Epigastric pain	S	36	8	16	5	46	12	6	1	53	5	6	1
	P	33	3	9	16	32	10	11	8	34	8	10	9
	Q	**0**	**0.78**	**1.51**	**2.62**	**0**	**1.25**	**2.14**	**2.96**	**0**	**1.47**	**1.98**	**2.93**

S = supplementation frequencies, P = placebo frequencies, and Q = normalized modalities quantify by MCA algorithm (in bold).

^*^MCA quantifications define scaled severity scores for each outcome.

**Table 4:** Intercept and slope arm differences (with *P* value, 95% CI) for primary and secondary outcomes over the 28 days of intervention.

	Intercept (u)	Intercept (u)	Intercept difference (u)	Slope (u)	Slope (u)	Slope difference (u)
	*P* value	*P* value	*P* value	*P* value	*P* value	*P* value
	[95% CI]	[95% CI]	[95% CI]	[95% CI]	[95% CI]	[95% CI]
	Supplementation	Placebo	Suppl. − placebo	Supplementation	Placebo	Suppl. − placebo
Primary outcome^*^	**1.195**	0.347	**+0.848**	−0.181	−0.258	+0.077
Functional dyspepsia	**0.017**	0.513	**<0.001**	0.619	0.504	0.542
	**[0.215; 2.174]**	[−0.692; 1.387]	**[−1.190; −0.505]**	[−0.893; 0.532]	[−1.015; 0.498]	[−0.326; 0.171]

Secondary outcomes^∧^						
Epigastric fullness	0.119	0.381	−0.263	−0.191	0.051	−**0.241**
	0.677	0.175	0.102	0.099	0.658	**<0.001**
	[−0.440; 0.678]	[−0.170; 0.932]	[−0.578; 0.053]	[−0.418; 0.036]	[−0.173; 0.274**]**	**[**−**0.369; **−**0.114]**
Bloating	**0.709**	**1.145**	−**0.437**	−0.133	0.034	−**0.167**
	**0.020**	**<0.001**	**0.011**	0.283	0.779	**0.017**
	**[0.110; 1.308]**	**[0.555; 1.735]**	**[**−**0.774; **−**0.099]**	[−0.376; 0.110]	[−0.205; 0.274]	**[**−**0.304; **−**0.030]**
Early satiety	−0.266	0.222	−**0.488**	−0.053	0.045	−0.098
	0.375	0.453	**0.004**	0.675	0.707	0.148
	[−0.855; 0.322]	[−0.358; 0.802]	**[**−**0.820; **−**0.157]**	[−0.289; 0.182]	[−0.188; 0.277]	[−0.231; 0.035]
Nausea	0.412	0.315	+0.096	−**0.290**	0.108	−**0.398**
	0.170	0.287	0.569	**0.030**	0.412	**<0.001**
	[−0.177; 1.001]	[−0.265; 0.895]	[−0.235; 0.428]	**[**−**0.552; **−**0.029]**	[−0.150; 0.365]	**[**−**0.545; **−**0.251]**
Vomiting	**0.625**	**0.948**	−0.322	0.214	**0.240**	−0.025
	**0.033**	**0.001**	0.051	0.056	**0.030**	0.688
	**[0.050; 1.200]**	**[0.381; 1.514]**	[−0.646; 0.002]	[−0.006; 0.434]	**[0.023; 0.456]**	[−0.149; 0.099]
Epigastric pain	**2.133**	**2.306**	−**0.174**	−**0.204**	−0.031	−**0.173**
	**<0.001**	**<0.001**	**0.022**	**0.036**	0.745	**0.002**
	**[1.869; 2.397]**	**[2.046; 2.566]**	**[**−**0.322; **−**0.025]**	**[**−**0.396;** −**0.013]**	[−0.220; 0.157]	**[**−**0.281; **−**0.065]**

In bold are shown the statistically significant evidences (*P* < 0.05).

^*^Estimated intercept and slope parameters (*P* value, 95% CI) of arm effects on functional dyspepsia (after 14 days and 28 days of treatment ) referred to variation (improving or not) from equal baseline conditions, adjusting for baseline values of the symptom severity scores (secondary outcomes) and dyspepsia typology. Therefore, patients reported their degree of functional dyspepsia variation on a symptomatic ordinal score (quantified by MCA), ranging from 0 (worsening or steadiness) to 3 (disappearance of symptoms).

^∧^Estimated intercept and slope parameters (*P* value, 95% CI) of arm effects on secondary outcomes, adjusted for dyspepsia typology, assessed using ordinal scores over time (baseline, after 14 days of treatment, and after 28 days of treatment). Patients reported their score (quantified by MCA), ranging from 0 (no symptoms) to 3 (severe symptoms).
